# Determinant of intention to receive COVID-19 vaccine among school teachers in Gondar City, Northwest Ethiopia

**DOI:** 10.1371/journal.pone.0253499

**Published:** 2021-06-24

**Authors:** Simegnew Handebo, Maereg Wolde, Kegnie Shitu, Ayenew Kassie

**Affiliations:** Department of Health Education and Behavioral Sciences, Institute of Public Health, College of Medicine and Health Sciences, University of Gondar, Gondar, Ethiopia; The Chinese University of Hong Kong, HONG KONG

## Abstract

**Background:**

Scientists across the world are working on innovating a successful vaccine that will save lives and end COVID-19 pandemic. World Health Organization (WHO) is working to make sure COVID-19 vaccines can be safely delivered to all those who need them. Indeed, the successful deployment and a sufficient uptake of vaccines is equally important. Acceptance and accessibility of such vaccine is a key indicator of vaccination coverage.

**Objective:**

This study aimed to assess the determinants of intention to receive COVID-19 vaccine among school teachers in Gondar City.

**Methods:**

An institution based cross-sectional study was conducted from December, 2020 to January, 2021. A total of 301 school teachers selected using stratified simple random sampling were included. Descriptive analysis such as medians, means, proportions, standard deviations and frequencies were computed. Linear regression analysis was done to identify factors associated with intention to receive COVID-19 vaccine. A p-value of less than 0.05 was used to declare statistical significance.

**Results:**

The median intention to receive COVID-19 vaccine was 3.33 with interquartile range of 2.67–4.0. Of the participants 54.8% had scored above the median of intention to receive COVID-19 vaccine score. 54% variance in intention to receive COVID-19 vaccine was explained by the independent variables. Being affiliated with other category of religion, bachelor degree educational status, perceived susceptibility, perceived benefit, perceived barrier, and cues to action were significantly associated with the intention to receive COVID-19 vaccine.

**Conclusion:**

The median score of intention to receive COVID-19 vaccine was 3.33. Socio-demographic and health beliefs influenced the intention to receive the COVID-19 vaccine in the study participant. Policy makers and stakeholders should focus on strong health promotion about risks of the pandemic, benefit, safety, and efficacy of vaccination.

## Introduction

The world is in the midst of a COVID-19 pandemic. World Health Organization (WHO) and partners are working on the response such as tracking the pandemic, advising on critical interventions, distributing vital medical supplies to those in need and now they are racing to develop and deploy safe and effective vaccines [1]. In the past 12 months after the beginning of the COVID-19 pandemic, several research teams are on developing vaccines that protect from SARS-CoV-2, the virus that causes COVID-19. The speed at which the first COVID-19 vaccines were developed was extraordinary. The development of a vaccine against COVID-19 has been much faster than the development of any other vaccine. Within less than a year several successful vaccines have already been announced and were approved for use in some countries [2].

Even though high-income countries comprising only 14% of the world’s population, they had made premarket purchase commitments to buy over half of all pre-sold doses as of 15 November 2020. Some high-income nations bought more doses than would be necessary to vaccinate their entire populations [3, 4]. As of 25 February 2021, 227.62 million doses of COVID‑19 vaccine have been administered worldwide based on official reports from national health agencies [2]. Yet there is no single low-income country has made a direct agreement to purchase vaccines [5]. The vaccines pillar of the World Health Organization’s Access to COVID-19 Tools (ACT) Accelerator, the COVAX Facility seeks to ensure a more equitable distribution of covid-19 vaccines, regardless of income levels [4, 5].

Despite this public perceptions and rumors on the vaccines like exaggerated claims on side effects may result in refusing vaccination. Such vaccine hesitancy is believed to be responsible for decreasing vaccine coverage and could lead to COVID‑19 outbreaks [6]. A global survey on a potential acceptance of a COVID-19 vaccine in 19 countries revealed that 71.5% of participants would likely take the vaccine if it was proven safe and effective. The acceptance rates vary between countries ranged from 90% (in China) to 55% (in Russia). The acceptance exceeded 80% in Asian nations (China, South Korea and Singapore). Higher trust in the information from government was correlated with the acceptance of a vaccine [7]. On the other hand, the acceptance of the COVID-19 vaccine was 67%, 77.6%, 85.8%, and 90.6% in the U.S., France, Australia, and Chile, respectively [8–11]. In Malaysia 48.2%, 29.8%, and 16.3% of the participants expressing a definite, probable, and possible intent to take the COVID-19 vaccine, respectively [12]. The other study in Saudi Arabia reported that 64.7% of participant have intention uptake the hypothetical COVID-19 vaccine [13]. From African countries the tendency toward acceptance of vaccine reaches from 81.6% in South Africa to 65.2% in Nigeria [7]. Such variation in willingness to accept a COVID-19 vaccine may result in difference in vaccine coverage and delay global control of the pandemic.

In a study done in seven European revealed 73.9% of the participant were willing to get vaccinated against COVID-19 if available. The willingness ranged from 62% in France to about 80% in Denmark and UK. The willingness to be vaccinated considerable vary across genders and age groups [14]. Similarly, a study in Italy showed 67% of health workers intended to be vaccinated. Younger age, who had close contact with high-risk groups, and received flu vaccination willing to get vaccinated [15].

Misinformation and lack of confidence in vaccines led to an insufficient uptake. Therefore, building the public confidence in vaccines is an important starting point towards acceptance and sufficient uptake of safe COVID-19 vaccines. In this regard, identifying and addressing possible gaps and COVID-19 vaccine related misconceptions in the public is a key element to be taken into consideration [16].

COVAX, the global effort working on equal access to COVID-19 vaccine, is estimated to cover the doses for about 20% Ethiopian population [5]. If this is the only amount of COVID-19 vaccine available to population in the country, vaccination may be distributed based on the risk level. Next to health professionals, school teachers would be the targets to receive the vaccine. There was no such study done in Ethiopia on the willingness to accept the COVID-12 vaccine. In addition, little is known about the predictors COVID-19 vaccine acceptance in the study area and at the country level. Therefore, the purpose of our study is to describe the current intention to accept COVID-19 vaccine among school teachers and identify the potential predictors.

## Methods

### Study design and setting

Institution based cross-sectional study was conducted among primary and secondary school teachers in Gondar city from December 2020 to February 2021. The city is located at about 727 kms away from Addis Ababa, the capital city of Ethiopia, and 180 km away from Bahir Dar, the capital city of Amhara Regional State. In 2019 the total population of the city is estimated to be 500,788, of whom 300,000 were men and 200,788 women.

### Population and sampling

The study participants were primary and secondary school teachers in Gondar city. Single population proportion formula was used to calculate the sample size with the following assumptions P (65.2%, proportion of intention to take vaccine among Nigerian people [7]), d (the permissible Margin of error 5%) and Zα/2 (the value of the standard normal curve score corresponding to the given confidence interval  =  1.96) corresponding to 95% confidence level. After adjusting for the total number study population and potential non-response rate, the final sample was 323.

Stratified simple random sampling technique was used to recruit the study participant. Firstly, stratification was done based on ownership of the schools into private and governmental. Then, four governmental and two private schools were selected using lottery method and the sample was proportionally allocated. Finally, study participants were selected randomly using computer-generated random numbers.

### Data collection procedure

The data were collected using pretested, structured, and self-administered questionnaire prepared by the investigators from different literature [12, 17, 18]. Firstly, the tool was prepared in English and then translated into the local Amharic language. Pretest was done on 5% of the total sample size and modifications were made accordingly. The tool consists socio-demographic characteristics, media exposure, health beliefs (perceived susceptibility, perceived severity, perceived benefit, perceived barrier, and cues to action), knowledge about COVID-19, preventive behaviors towards COVID-19, and intention to receive COVID-19 vaccine variables.

Trained public health professionals facilitate and supervised the data collection process. Safety precautions towards COVID-19 preventions were taken during the data collection process. Data collection facilitator and supervisors took one day training on the objective of the study, content of the questionnaire, and ethical issues need to be taken during the data collection process. Each returned instrument was reviewed for completeness and consistency on daily basis.

### Measurements

#### Perceived susceptibility

Refers to a person’s subjective perception of the risk of acquiring COVID-19 and measured by three five-point Likert scale items. The higher summed score indicates higher perceived susceptibility towards COVID-19 [17, 18].

#### Perceived severity

Refers to a person’s perception on the seriousness of contracting COVID-19 and measured by three five-point Likert scale items. The higher summed score indicates higher perceived severity of COVID-19 [17, 18].

#### Perceived benefit

Refers to a person’s perception of the effectiveness of COVID-19 vaccine to prevent COVID-19 and measured by three five-point Likert scale items. The higher summed score indicates higher perceived benefits of COVID-19 vaccine [17, 18].

#### Perceived barriers

Refers to a person’s perception on the obstacles to receive COVID-19 vaccine and measured by five items with five-point Likert scale. The higher summed score indicates higher perceived barriers to restrict receiving COVID-19 vaccine [17, 18].

#### Cues to action

Refers to stimulus needed to trigger the decision-making process to accept COVID-19 vaccine and measured by three five-point Likert scale items. The higher summed score indicates the impact of cues to receive COVID-19 vaccine [17, 18].

#### Knowledge about COVID-19

Refers to participant’s awareness about COVID-19 and measured by 11 items with three response categories (1 = True, 2 = False and 3 = I don’t know). A correct answer was coded as 1 point whereas, the incorrect and unknown answers were recoded to zero. The higher summed score indicates higher knowledge about COVID-19 disease.

#### Preventive health behaviors

Refers to the handwashing, physical distancing, and facemask wearing practices to prevent COVID-19 infection and measured by five items with five-point Likert scale. The higher summed score indicates compliance of the participant with COVID-19 preventive practices.

#### Intention to receive COVID-19 vaccine

Refers to a person’s readiness to receive COVID-19 vaccine and measured by three items with five-point Likert scale. The higher summed score indicates higher intention to receive COVID-19 vaccine.

### Data processing and analysis

Data were coded and cleaned for completeness and consistency. Then, entered in to EpiData version 4.6 and exported into to STATA version 14 statistical software for analysis. Descriptive analysis like medians, means, proportions, standard deviations, inter quartile range, and frequencies were computed. Spearman’s rank correlation was done see the relation between health beliefs, knowledge about COVID-19, preventive behavior, and intention to receive COVID-19. The test of homoscedasticity was conducted with the predictors and the result support the assumption. Linearity was checked using scatter plot of the standardized residuals versus the predicted values from the regression analysis. Multicollinearity was checked using variance inflation factors (VIF) and the value of all variables was less than 5. But we Simple linear regression analysis was computed and all independent variables with p-value less than 0.25 were entered in multiple linear regression. R-square was used to assess the variation in intention to receive COVID-19 vaccine explained by the independent variables. An unstandardized β coefficient was used to interpret the effect of the predictors. A P-value less than 0.05 was used for declaring statistical significance.

### Ethical considerations

Ethical clearance was obtained from the Institutional Review Board of the University of Gondar a reference number of V/P/RCS/05/568/2020. Letter of permission was obtained from Gondar city administrative education office. After the purpose and objective of the study have been informed, written consent was obtained from each study participant. In order to keep confidentiality, all identifiers of the study participants were not recorded.

## Results

A total of 301 school teachers were participated in this study with a response rate of 93.2%. The mean age of participants was 39.5 (SD ± 8.7 years) with the range of 21 to 64 years. More than half (59.1%) of the participants were males. The majority (83.4%) of them were affiliated with Ethiopian Orthodox Church. Most of them (63.5%) were from public schools (Table 1).

**Table 1:** Socio-demographic characteristics of school teachers in Gondar city, North West Ethiopia, 2021 (n = 301).

Variables	Category	Female n (%)	Male n (%)
Age	Less than 39	74 (49.33)	76 (50.67)
	Above 39	49 (32.45)	102 (67.55)
Educational status	Diploma	38 (63.33)	22 (36.67)
	Degree	76 (41.53)	107 (58.47)
	Masters	9 (15.52)	49 (84.48)
Religion	Orthodox	109 (43.43)	142 (56.57)
	Muslim	8 (25.81)	23 (74.19)
	Others	6 (31.58)	13 (68.42)
Family size	Less than 4	70 (42.94)	93 (57.06)
	Greater than 4	53 (38.41)	85 (61.59)
Monthly income	Less than 5500 ETB	41 (53.95)	35 (46.05)
	5500–6900 ETB	39 (50.65)	38 (49.35)
	Above 6900 ETB	43 (29.05)	105 (70.95)
School type	Private	46 (41.82)	64 (58.18)
	Pubic	77 (40.31)	114 (59.69)
Chronic disease*	Yes	24 (42.11)	33 (57.89)
	No	99 (40.57)	145 (59.43)
Perceived health condition	Healthy	100 (39.06)	156 (60.94)
	Non-healthy	23 (51.11)	22 (48.89)
Listening radio	No	47 (55.29)	38 (44.71)
	Yes	76 (35.19)	140 (64.81)
Watching television	No	2 (14.29)	12 (85.71)
	Yes	121 (42.16)	166 (57.84)
Reading magazines/ newspapers	No	53 (49.53)	54 (50.47)
	Yes	70 (36.08)	124 (63.92)

*diabetics, hypertension, asthma and HIV/AIDS.

### COVID-19 related knowledge and preventive practice

The reliability test of COVID-19 related knowledge items for Cronbach’s alpha was 88.0% The median COVID-19 related knowledge score of the participant was 9 with an inter quartile range of 3. According to the Blooms cut-off point one third (33.2%) of the participant had moderate knowledge about COVID-19. Regarding COVID-19 preventive practices, only 8.64%, 14.29%, and 9.97% of the respondents had kept their physical distance, washed their hands frequently for at least 20 minutes, and wore facemask as recommended, respectively. Significant number of teachers 43.52%, 31.23%, and 32.89% reported that they were rarely kept their physical distance, washed their hand frequently for at least 20 seconds, and wore facemask, respectively (Table 2).

**Table 2:** COVID-19 preventive behaviors among school teachers in Gondar city, Northwest Ethiopia 2021(n = 301).

Items	Response categories			
	Rarely	Some times	Often	Always
I keep a distance of at least two meters from others.	131(43.52)	90 (29.90)	54 (17.94)	26 (8.64)
I place a tissue paper or bending elbow in front of my mouth and nose when coughing or sneezing.	60 (19.93)	75 (24.92)	83 (27.57)	83 (27.57)
I wash my hands regularly with soap and water for at least 20 seconds every hour.	94 (31.23)	79 (26.25)	85 (28.24)	43 (14.29)
I do not touch my eyes, nose and mouth by unwashed hands.	84 (27.91)	94 (31.23)	86 (28.57)	37 (12.29)
I wear facemask consistently whenever I go out to my home.	99 (32.89)	101(33.55)	71 (23.59)	30 (9.97)

### Intention to receive COVID-19 vaccine

The intention to receive COVID-19 vaccine was assessed using three five-point Likert scale items with the reliability test of Cronbach’s alpha 90.0%. Since intention to receive COVID-19 vaccine score was not normally distributed, we computed the median and inter-quartile range. The median intention to receive COVID-19 vaccine was 3.33 with interquartile range of 2.67–4.0. More than half (54.8%) of the participants had scored above the median of intention to receive COVID-19 vaccine score.

### Health beliefs about COVID-19

Health beliefs about COVID-19 of participants were measure using constructs of health belief model; perceived susceptibility, perceived severity, perceived benefit, perceived barrier, and cues to action. The reliability test of health belief items for Cronbach’s alpha ranged from 65.0% for cues to action to 87.0% of perceived benefit. **Table 3** presented the detail distribution of health belief variables.

**Table 3:** Distribution of health belief on intention to receive COVID-19 vaccine among school teachers in Gondar city, North West Ethiopia, 2021.

Construct domain	Number of items	Minimum	Maximum	Median	IQR	Cronbach’s Alpha
Perceived susceptibility	3	1	5	3.33	1.67	0.78
Perceived severity	3	1	5	3.33	1.33	0.79
Perceived benefit	3	1	5	3.33	1.33	0.87
Perceived barrier	5	1	5	3.20	1.20	0.73
Cues to action	3	1	5	3.33	1.33	0.65
Knowledge about COVID-19	11	0	0.91	0.82	0.27	0.88
COVID-19 Preventive behavior	5	1	4	2.20	1	0.78
Intention to receive vaccine	3	1	5	3.33	1.33	0.90

IQR = Interquartile range.

### Correlation between health beliefs, knowledge, preventive behavior, and intention to receive COVID-19 vaccine

The Spearman’s rank correlation analysis was executed to assess the relationship between health beliefs, knowledge, preventive behavior, and intention to receive COVID-19 vaccine. Intention to receive COVID-19 vaccine showed a significant positive correlation with health beliefs, knowledge, and preventive behaviors. Perceived benefit showed a significant positive correlation with other health beliefs, knowledge, preventive behavior, and intention to receive the vaccine. Perceived benefit showed strongest correlation with intention to receive COVID-19 vaccine and weakest correlation with COVID-19 preventive behavior. Intention to receive COVID-19 vaccine showed weakest correlation with perceived barrier and COVID-19 preventive behavior (Table 4).

**Table 4:** Spearman’s rank correlation of health belief on intention to receive COVID-19 vaccine among school teachers in Gondar city, North West Ethiopia, 2021.

Constructs	PSU	PSE	PBE	PBA	CA	KG	CPB	IN
Perceived susceptibility (PSU)	1.00							
Perceived severity (PSE)	0.60*	1.00						
Perceived benefit (PBE)	0.43*	0.59*	1.00					
Perceived barrier (PBA)	0.36*	0.35*	0.35*	1.00				
Cues to action (CA)	0.29*	0.40*	0.49*	0.45*	1.00			
Knowledge about COVID-19 (KG)	0.17*	0.26*	0.25*	0.06	0.15*	1.00		
COVID-19 preventive behavior (CPB)	0.04	0.17*	0.14*	-0.04	0.09	0.12*	1.00	
Intention to take vaccine (IN)	0.40*	0.48*	0.60*	0.16*	0.46*	0.22*	0.15*	1.00

* Significant correlation coefficient at p value < 0.05.

### Predictors of intention to receive COVID-19 vaccine

Variables with p-value less than 0.2 in simple linear regression were entered to multiple linear regression. Sociodemographic variables (age, religion, educational status, perceived health condition, and presence chronic disease), media exposure (listening radio and watching television), health beliefs (perceived susceptibility, perceived severity, perceived benefit, perceived barrier, and cues to action), knowledge about COVID-19, and preventive behaviors towards COVID-19 were candidate for multiple linear regression at p value <0.2.

In multiple linear regression, the predictors explained nearly 54% variance in intention to intention to receive COVID-19 vaccine. Being affiliated with other category of religion (β = 1.18; 95% CI: 0.13, 2.23), bachelor degree educational status (β = -1.23; 95% CI: -1.88, -0.59), perceived susceptibility (β = 0.16; 95% CI: 0.05, 0.27), perceived benefit (β = 0.38; 95% CI: 0.27, 0.48), perceived barrier (β = -0.16; 95% CI: -0.24, -0.08), and cues to action (β = 0.34; 95% CI: 0.22, 0.45) were significantly associated with the intention to receive COVID-19 vaccine at 5% level of significance (Table 5).

**Table 5:** Multiple linear regression of intention to receive COVID-19 vaccine among school teachers in Gondar city, Northwest Ethiopia, 2021.

Variable		Unstandardized B	Standardized β	95% CI for B	P-value
Age**		0.01	0.04	-0.02, 0.04	0.41
Religion	Orthodox (ref.)				
	Muslim	0.08	0.01	-0.75, 0.90	0.852
	Others	1.18*	0.09	0.13, 2.23	0.028
Educational status	Diploma (ref.)				
	Degree	-1.23*	-0.20	-1.88, -0.59	0.000
	Masters	-0.79	-0.10	-1.59, 0.003	0.051
Chronic disease	Yes (ref.)				
	No	-0.53	-0.07	-1.31, 0.26	0.187
Perceived health condition	Healthy (ref.)				
	Non-healthy	-0.33	-0.04	-1.17, 0.52	0.450
Listening radio	No (ref.)				
	Yes	0.21	0.03	-0.34, 0.76	0.455
Watching television	No (ref.)				
	Yes	-0.003	-0.0002	-1.22, 1.21	0.996
Perceived susceptibility**		0.16*	0.16	0.05, 0.27	0.003
Perceived severity**		0.13	0.12	0.0001, 0.25	0.05
Perceived benefit**		0.38*	0.38	0.27, 0.48	0.000
Perceived barrier**		-0.16*	-0.21	-0.24, -0.08	0.000
Cues to action**		0.34*	0.30	0.22, 0.45	0.000
Knowledge about COVID-19**		0.03	0.03	-0.07, 0.13	0.536
COVID-19 Preventive behavior**		0.026	0.03	-0.04, 0.09	0.447
Constant		2.51		0.16, 4.87	

* Significant coefficient at p value <0.05

**continuous variable, ref. = reference.

## Discussion

This research assessed the predictors of intention to receive COVID-19 vaccine among school teachers in Gondar city, North West Ethiopia. In the study COVID-19 related beliefs based on health belief model constructs were also fitted as a predictor of intention to receive COVID-19 vaccine. The predictors in the regression model explained nearly 54% variance in intention to receive COVID-19 vaccine. Being affiliated with other category of religion, perceived susceptibility, perceived benefit, and cues to action positively associated with the intention to receive COVID-19 vaccine. Instead, having bachelor degree educational status and perceived barrier were negatively associated the intention to receive COVID-19 vaccine.

Vaccine acceptance seems to have a decisive role for successful control of the COVID-19 pandemic [19]. In this study, 54.8% of the participants had above the median score of intention to accept the vaccine. This study result is lower than vaccination uptake for herd immunity, which would need to be at least 67% [20]. This finding is also lower that previous studies done in U.S., France, Australia, and Chile Malaysia, Saudi Arabia, seven European countries, and health workers in Italy [7–15]. Similarly, vaccine acceptance is relatively higher among population in South Africa and Nigeria [7]. The findings remind the health sector and those planning on vaccine distribution that more efforts are needed to promote COVID-19 vaccine acceptance and uptake. This may go beyond undertaking educational campaigns that raise awareness to aspiring behavior changes by improving knowledge and attitude [21].

We analyzed factors associated with intention to receive COVID-19 vaccine. From socio-demographic factors religious affiliation and educational status were associated with intention to accept the vaccine. Compared to individuals affiliated with Orthodox religion, those affiliated with other category of religions (i.e., Catholic and Protestant) had increased intention to accept the vaccine. This may be attributed to perceived lower risk to get infected or vaccine hesitancy due to religious values or lack of trust in the health system. This informs us the need to engage religious leaders and communities from the very beginning in designing vaccine promotion strategies [21]. Although educated individual are aware of the health benefit of vaccine, in this study participants who achieved university degree had a decreased intention to receive the vaccine compared to those who received college diploma. Different findings were reported by studies done in 19 countries at global level and US where, increase in years of education was associated with increased acceptance of the COVID-19 vaccine [7, 8]. In Australia reluctance to be vaccinated against COVID-19 related with lower education level [10]. This may be attributed to substantial misinformation spread through multiple channels about the vaccine [22].

Intention to get vaccinated is the result of a combination of different factors including perceived risk and severity of the infection [21]. In the present study, from health beliefs based on the constructs of HBM perceived susceptibility, perceived benefit, and cues to action had a significant and positive association with intention to accept COVID-19 vaccine. This indicates that if people perceive that they are at high risk of contracting COVID-19, or that the vaccine effectively prevent the disease, or messages/stimulus triggered to accept COVID-19 vaccine, they will be more willing to get vaccinated [12, 21]. The findings of this study suggest that perceived benefit and cues to action are the most important predictor of intention to accept COVID-19 vaccine. Similar study findings were reported by a study done in Malaysia. Henceforth, increasing the perception of the benefits of vaccination, susceptibility to infection, and message triggering acceptance of the vaccine need to be a focus of information, education and communication along with vaccine distribution and coverage [12]. A campaign on the social benefits of vaccination enhance the willingness to be vaccinated [14].

On the other hand, perceived barrier had a significant and negative association with intention to accept COVID-19 vaccine. This mean that individuals with high perceived barrier less likely intend to receive the vaccine. The perceived barrier found in this study were worry about side effects, efficacy, safety, and cost of the COVID-19 vaccine. Similar finding is reported by study done in Malaysia [12]. Most of the time such risk assessments are based on personal experience or rumors from different sources, significantly decreases the intention of people to accept the vaccination [21]. This may infer the importance of more intensively disseminating relevant information emphasizes on the safety and effectiveness of the vaccination.

To the best of the investigators’ knowledge, this is the first studies that tries to address the intention to accept COVID-19 vaccine among at risk group in Ethiopia. There are few limitations in the present study. First, the data on intention to receive COVID-19 vaccine was self-reported and may be subject to social desirability bias. The other is that the participants were asked to report their intention to receive the COVID-19 vaccine if it is available. This intention may be changed actually when the vaccine is readily available. Finally, as noted earlier this study was cross-sectional by design, which makes it difficult to identify a cause-and-effect association between the variables.

## Conclusion

Our study found 54.8% of the participants scored above the median of intention to receive COVID-19 vaccine score. When an effective COVID-19 vaccine is available above half of the study sample have intention to accept the vaccine. However, socio-demographic and health beliefs that influences the intention to receive the vaccine need to be carefully addressed. Policymakers and stakeholders should focus work on open and transparent messaging about risks of the pandemic, benefit, safety, and efficacy of vaccination. Evidence-based communication, building trust, and working in partnership with influential member of the community are key strategies to improve uptake of the vaccine and break the transmission of the disease.
